# Non-invasive and invasive diagnoses of aspergillosis in a rat model by mass spectrometry

**DOI:** 10.1038/s41598-017-16648-z

**Published:** 2017-11-28

**Authors:** Dominika Luptáková, Tomáš Pluháček, Miloš Petřík, Jiří Novák, Andrea Palyzová, Lucie Sokolová, Anton Škríba, Blanka Šedivá, Karel Lemr, Vladimír Havlíček

**Affiliations:** 1 Institute of Microbiology of the Czech Academy of Sciences, Prague 4, 142 20 Czech Republic; 2 Regional Centre of Advanced Technologies and Materials, Department of Analytical Chemistry, Olomouc, 771 47 Czech Republic; 3 Institute of Molecular and Translational Medicine, Palacky University, Olomouc, 779 00 Czech Republic; 4 University of West Bohemia, Plzen, 306 14 Czech Republic

## Abstract

Invasive pulmonary aspergillosis results in 450,000 deaths per year and complicates cancer chemotherapy, transplantations and the treatment of other immunosuppressed patients. Using a rat model of experimental aspergillosis, the fungal siderophores ferricrocin and triacetylfusarinine C were identified as markers of aspergillosis and quantified in urine, serum and lung tissues. Biomarkers were analyzed by matrix-assisted laser desorption ionization (MALDI) and electrospray ionization mass spectrometry using a 12T SolariX Fourier transform ion cyclotron resonance (FTICR) mass spectrometer. The limits of detection of the ferri-forms of triacetylfusarinine C and ferricrocin in the rat serum were 0.28 and 0.36 ng/mL, respectively. In the rat urine the respective limits of detection achieved 0.02 and 0.03 ng/mL. In the sera of infected animals, triacetylfusarinine C was not detected but ferricrocin concentration fluctuated in the 3–32 ng/mL range. Notably, the mean concentrations of triacetylfusarinine C and ferricrocin in the rat urine were 0.37 and 0.63 μg/mL, respectively. The MALDI FTICR mass spectrometry imaging illustrated the actual microbial ferricrocin distribution in the lung tissues and resolved the false-positive results obtained by the light microscopy and histological staining. Ferricrocin and triacetylfusarinine C detection in urine represents an innovative non-invasive indication of *Aspergillus* infection in a host.

## Introduction

More than 1.6 million people are estimated to die of fungal diseases each year and about a billion people have cutaneous fungal infections^1^. The most common and life-threatening infections are cryptococcosis, candidiasis, aspergillosis, blastomycosis, histoplasmosis, coccidioidomycosis, and pneumocystosis^2^. Fungal infection-related deaths in AIDS patients are estimated to be more than 700,000 cases (47% of all HIV-associated deaths) annually^3^.

Currently, invasive pulmonary aspergillosis (IPA) alone accounts for 450,000 deaths per year^2^. IPA complicates cancer chemotherapy, particularly in patients with leukemia, transplantations and the treatment of patients with other immunosuppressed conditions, including patients with HIV infection. The mortality rates range from 40% to 90% in high-risk populations and are dependent on factors such as the host immune status, the site of infection, and the treatment regimen applied^4^. Scenarios in which avoidable deaths can be reduced have been modeled until 2020 using published outcomes of the real-life impact of diagnostics and generic antifungal drugs^3^. The annual death rates could be reduced if early and reliable diagnostic tools are available. In addition to IPA, there are millions of annual cases of allergic bronchopulmonary aspergillosis (ABPA), which may advance to chronic pulmonary disease requiring antifungal therapy^5^. The primary route of human infection is via the inhalation of airborne conidia, followed by conidia deposition in the bronchioles or alveolar spaces. The average person is estimated to inhale up to 200 *A. fumigatus* conidia per day^6^. In IPA-susceptible patient populations, the mucosal defense mechanism in the lung is compromised, leading to fungal colonization and growth. By contrast, in healthy individuals, conidia that are not removed by mucociliary clearance mechanisms encounter epithelial cells and alveolar macrophages that are responsible for phagocytosis and the recruitment of infiltrating neutrophils that kill *Aspergillus* conidia^6^.

A standard *Aspergillus* diagnostic approach involves the culture of bronchoalveolar lavage samples; this method is sensitive but is invasive and time-consuming. Another invasive but faster diagnostic approach involves the typing of the fungal ribosomal protein equipment using matrix-assisted laser desorption ionization (MALDI) profiling, which requires two- to three-day-old cultures, representing a delay that immunosuppressed patients cannot afford^7^. Commercial tools based on DNA typing/PCR or galactomannan serology tests have a lower predictive value, specificity and sensitivity^8^. In (1–3)-*β*-D-glucan (BG) testing, serology levels exceeding 80 pg/mL are considered positive for IPA. However, the *Candida*, *Fusarium* and *Pneumocystis* species also produce BG. Another study based on gliotoxin detection in human serum was reported by Cerquieira *et al*.^9^. In that study, the lower limit of quantitation (LLOQ) using a triple quadrupole mass spectrometer was 10 ng/mL in the multiple reaction monitoring mode, and gliotoxin was detected in only eight of thirty samples obtained from patients at risk of invasive aspergillosis. Therefore, even at the beginning of the 21^st^ century, a sensitive non-invasive tool for the early detection and diagnosis of aspergillosis remains lacking^10^. Furthermore, distinguishing between colonization (e.g., in ABPA or cystic fibrosis patients) and invasion (IPA) is challenging.

Recently, we described fungal siderophores as specific markers of fungal infections^11^; siderophores are iron chelators with binding constants of 10^20^–10^50^ that are capable of extracting iron, e.g., from host transferrin^12^. Siderophore biosynthesis is important for the virulence of not only *A. fumigatus* but also of other pathogenic microbes, such as *Yersinia pestis*, *Mycobacterium tuberculosis*, *Bordetella pertussis*, *Scedosporium apiospermum* and *Pseudomonas aeruginosa* ^13^. In our previous *ex vivo* study, we described the fungal burden in rat lungs using elemental mass spectrometry imaging (MSI)^13^. The iron distributions derived from the fungal siderophores and the silver or gold distributions derived using a similar sample staining protocol but specifically incorporated into fungal bodies were used as elemental disease descriptors. The limit of detection was determined to be 30 ng of Au/1 g of lung tissue (with a 5 μm laser focus). At the molecular level, another research group detected two siderophores using desorption electrospray mass spectrometry in the wings of bats suffering from white nose syndrome^14^. Both siderophores, i.e., triacetylfusarinine C (TAFC) and ferrichrome, were produced by *Pseudogymnoascus destructans*. In a clinical study, TAFC was also detected in human sera from patients at risk of invasive aspergillosis. An LOQ of 5 ng/mL was reported in the multiple reaction monitoring mode using a triple quadrupole mass spectrometer^15^.

In our current work, we provide molecular quantitative data indicating that *Aspergillus* siderophores are effectively secreted in urine during microbial infection and define these siderophores as non-invasive biomarkers of aspergillosis. The siderophore frequencies can be much higher than the frequencies of other small molecules that are currently defined as *Aspergillus* virulence factors, including gliotoxin^12^. We compared our quantitative results of the distribution of TAFC with that of another *Aspergillus* siderophore, i.e., ferricrocin (FC), in rat serum and lung tissues and showed that these biomarkers are highly sensitive. Furthermore, we report for the first time the molecular MALDI-MSI of these disease biomarkers in lung tissues. The use of Continuous Accumulation of Selected Ions (CASI) using a Fourier transform ion cyclotron resonance (FTICR) mass spectrometer provided a low detection limit of the siderophores. The structural assessments^16^ and dereplication of the siderophores (i.e., the classification of already known siderophores) from imzML data and high-performance liquid chromatography (HPLC) FTICR datasets were facilitated by our in-house *de novo* free dereplication tool, i.e., CycloBranch^17,18^.

## Results and Discussion

### Monitoring the extent of fungal burden in lung tissues

Independent imaging tools were used to monitor the development of aspergillosis. *In vivo* imaging was performed using positron emission tomography (PET) and computed tomography (CT). Although CT scans of the infected lungs indicated a pathology of an unknown etiology (non-physiological gray signs potentially revealing the presence of a tumor, inflammation or infection), the fused PET/CT images enabled us to observe the locations where ^68^Ga-doped TAFC was selectively captured by the fungus (Supplementary Fig. S1).

**Figure 1:**
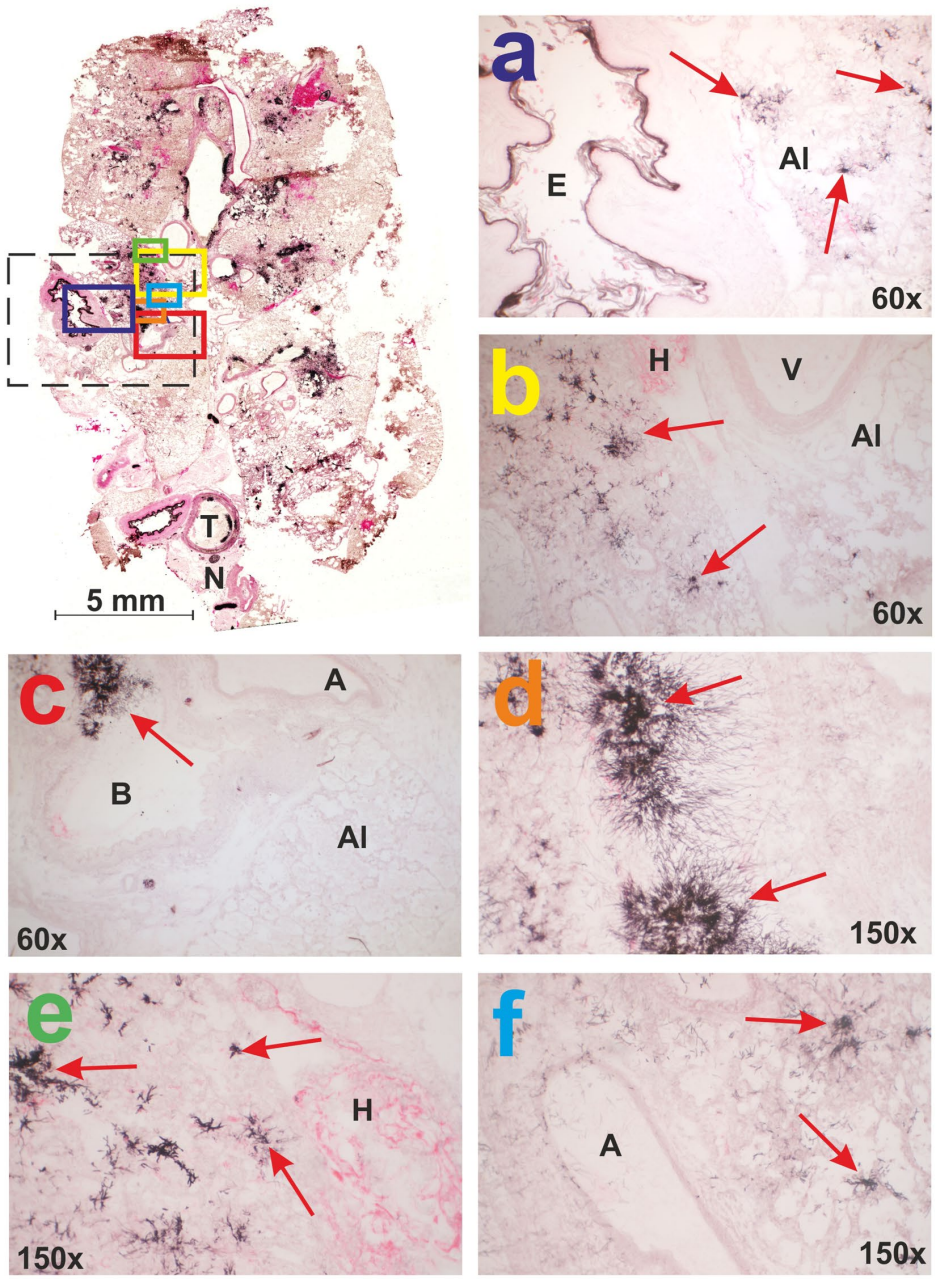
Histological examination of rat lung tissue infected with *Aspergillus fumigatus*. GMS and eosin staining was used for the area definition, which was examined in parallel by MALDI-MSI (dashed line in black). Fungal hyphae are marked by red arrows in all panels (a–f). Descriptors: A (arteriole), Al (alveoli), B (primary bronchus or bronchiole), E (esophagus), H (hemorrhage), T (trachea), V (vein), N (nervus vagus).

Grocott’s methenamine silver (GMS) and eosin staining was performed as another independent *ex vivo* tool and revealed extensive fungal burden in the infected animals, which was manifested by dense hyphae growth from the cell walls into the lumen of the trachea, bronchi/bronchioli and alveoli. Optical microscopy of the GMS and eosin-stained samples revealed the presence of intense black-colored *Aspergillus* hyphae in all infected biological replicates (Fig. 1a). Upon the application of the intratracheal spore suspension, the hyphae were present throughout the respiratory tract from the trachea to the terminal alveoli (Fig. 1b). Both sides were typically affected, although the spores were doped only on a single side. The hyphae penetrated the submucosal layer and ciliated epithelium towards the tracheal lumen. Fungal bodies also grew in the bronchioles, forming compacted assemblies (Fig. 1c,d). In the alveolar ducts and alveoli, the infection affected both the cell walls and the alveolar space and induced clogging and collapse of the lung tissue. We speculate that the lung collapse was the primary cause of host death. Hemorrhage was often observed in areas affected by *Aspergillus* (Fig. 1e). The hyphae were also detected in the veins and arteries near the infected bronchioles (Fig. 1f).

### Quantification of the siderophores in rat serum

**Figure 2:**
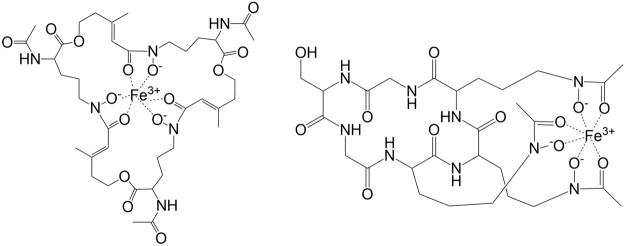
Structures of Fe-TAFC (left) and Fe-FC (right).

Upon lung inoculation, the immune system responds to the fungal growth and partially degrades the growing mycelia. Siderophore synthesis and iron uptake are *Aspergillus* virulence factors that occur at high frequencies^19^. *A. fumigatus* utilizes the following two systems for iron acquisition: siderophore-mediated iron uptake and reductive iron assimilation^20^. The fungus produces four known siderophores^21^. Fusarinin C and TAFC are excreted to chelate extracellular iron, whereas FC and hydroxy-ferricrocin are involved in hyphal and conidial intracellular iron storage. Using our rat model, we detected and monitored extracellular TAFC and intracellular FC (Fig. 2).

During infection in an immunocompromised host, the defense capacity is rapidly exhausted, and an encapsulated aspergilloma is formed. The growing fungal balls penetrate the lung wall, enabling the further transfer of fungal secondary metabolites into the blood. The binding of both siderophores to transport proteins has been reported to be low^22^. FC binds serum proteins at a higher capacity than TAFC, suggesting that its clearance from serum could be slower. Indeed, in serum from six infected animals, we detected ferri-ferricrocin (Fe-FC) concentrations in the range of 3–32 ng/mL, which contrasted with ferri-triacetylfusarinine C (Fe-TAFC) concentrations that were below the detection limit (Supplementary Fig. S2). The mean value of Fe-FC was 12.52 ± 4.44 ng/mL (Supplementary Table S2). No siderophores were detected in the control animals. In our HPLC-electrospray ionization (ESI)-CASI-Fourier Transform Mass Spectrometry (FTMS) setup, the limits of detection (LODs) of Fe-TAFC and Fe-FC in the serum samples were 0.28 and 0.36 ng/mL, respectively (Supplementary Figs S3 and S4). Galactomannan (GM) results (Platelia Aspergillus EIA, Bio-Rad, Prague, Czech Republic) on the same serum samples confirmed the pathogen presence in all infected samples. One control sample (S14) was false positive (Supplementary Fig. S2, Panel B). In 1,3-*β*-D-glucan tests (Fungitell, Associates of Cape Cod, Falmouth, USA) all infected rat sera revealed 1,3-*β*-D-glucan concentrations >523 pg/mL. Two control samples were false positive (Supplementary Fig. S2, Panel C).

In previous work by Carrol *et al*., the reported TAFC concentration in human serum was in the ng/mL range^15^. When the sodiated and potassiated Fe-TAFC forms were monitored, the LOD in a multiple reaction monitoring experiment using a triple quadrupole instrument was determined to be 1 ng/mL. With a 0.1 Da extraction window, considerable chemical noise could cause false-positive findings; even a healthy individual could show a low TAFC level, which obviously cannot be the case. In our high mass resolution CASI experiment, better selectivity was achieved with a 0.01 Da width for ion chromatogram extraction. We obtained clean chromatograms at siderophore concentration 0.1 ng/mL (Supplementary Fig. S5).

### Towards non-invasive diagnoses of aspergillosis

**Figure 3:**
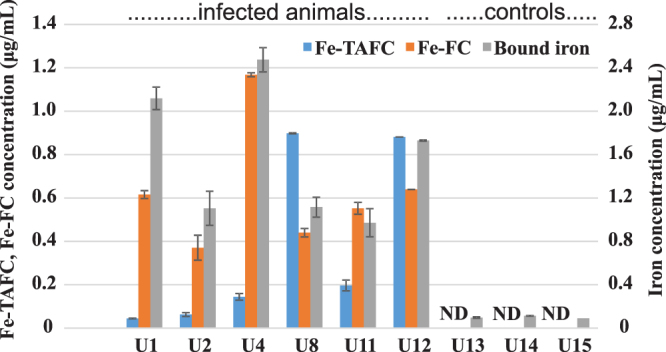
Profiling of Fe-TAFC and Fe-FC in urine from infected (left six) and control (right three) animals. Data were generated by liquid chromatography and CASI-FTICR mass spectrometry. Bound iron concentration data were obtained using solution ICP-MS.

Microbial siderophores are small molecules that are not captured by the kidney and can be effectively secreted in urine. Indeed, Fe-TAFC and Fe-FC were detected in urine from all infected animals (Fig. 3). Compared to the serum samples, the concentration levels of the siderophores were much higher in urine, reaching concentrations as high as 1 μg/mL. The mean concentration values of Fe-TAFC and Fe-FC were 0.37 ± 0.17 and 0.63 ± 0.12 μg/mL, respectively. Statistical significance was confirmed by z-test for both molecules (Supplementary Table S5). In the rat urine the respective limits of detection were lower than those in serum and achieved 0.02 and 0.03 ng/mL, respectively.

Notably, all specimens (i.e., urine, serum, lung tissue) from all biological replicates were collected simultaneously on day 3 post-infection. In two animals (U8 and U12), the extracellular TAFC dominated the intracellular FC. In the other four animals, the mutual siderophore ratio was the opposite. Therefore, none of these biomarkers can be distinguished as “early” or “late” with respect to the cellular location of the siderophore. In our rat model, the occurrence of Fe-FC is clearly more dominant than the concentration of Fe-TAFC. The mutual ratio can be affected not only by possible protein binding but also by the genetic equipment of the corresponding fungal producer. However, no significant difference was detected between the Fe-TAFC and Fe-FC concentrations at the 0.05 significance level (Supplementary Table S6).

By contrast, the molecular detection of microbial siderophores in mammalian urine provides clear evidence of microbial infection in the host. The actual concentration can be a potential measure of the disease severity. The significant molecular specificity and sensitive detection limits make our method a good candidate for transfer to human diagnostics. LC-CASI-FTMS monitoring has the potential to be used in future antifungal treatment monitoring, such as in patients suffering from chronic aspergillosis. Although the LC-CASI-FTMS approach is costly in terms of the required instrumental equipment, this approach is one of the most sensitive and specific approaches developed to date that is capable of solving this task.

Analogous to the molecular markers, a similar trend was observed at the elemental level (Fig. 3). The inductively coupled plasma mass spectrometry (ICP-MS) measurements revealed much higher distributions (1.58 ± 0.25 μg/mL) of bound iron in infected animals than those in the controls (0.09 ± 0.01 μg/mL). This observation correlates well with our previous results of laser ablation ICP-MS of infected lung tissues, which also revealed similar chemical noise^13^.

### Molecular description of invasive aspergillosis in lung tissues

MALDI-CASI-FTICR mass spectrometry can be a specific molecular imaging tool for describing an infectious disease in rat lung tissue, resolving possible false-positive results obtained from light microscopy. To the best of our knowledge, this report is the first of a true molecular MSI application in infectious disease diagnostics. The lungs are a low-density aerial tissue dominated by protonated or sodiated phosphocholines, namely, PC(32:0) and PC(34:1), in which the siderophore signals are buried (Supplementary Fig. S6).

**Figure 4:**
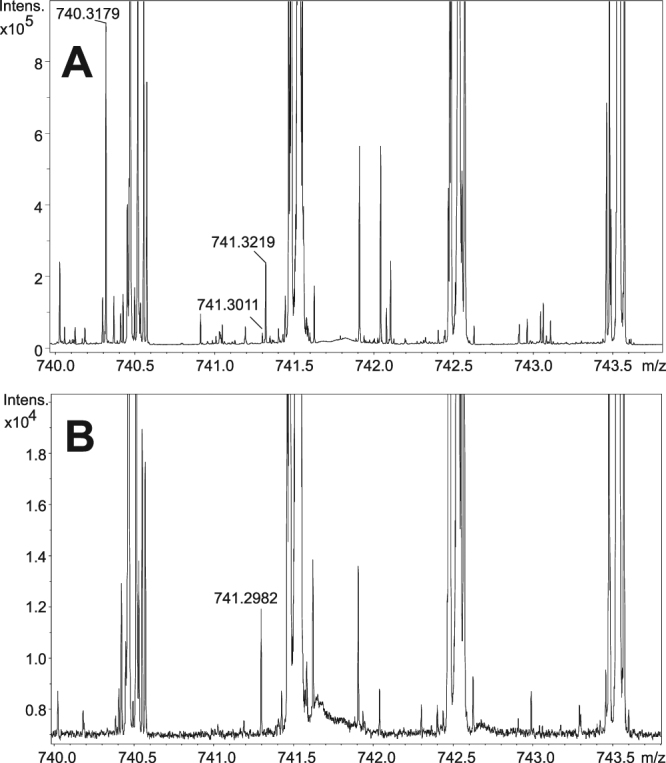
MALDI-CASI-FTICR mass spectrum of infected lung tissue with [FC + Na]^+^ ion signals (Panel A). Isotopic A + 1 ion is also labeled (m/z 741.3219). MALDI mass spectrum of control tissues does not reveal any fungal biomarkers (Panel B).

To examine the siderophore distribution in the rat lung sections, we analyzed the area indicated in Fig. 1. Interestingly, the siderophore desferri-forms were the most abundant species in the MALDI-CASI-FTICR-MSI analysis. Notably, a low abundance of [M + Na]^+^ ions of FC was observed at m/z 740.3179, and the presence of many other species with different elemental compositions and mass defects were detected at the same (740) nominal mass (Fig. 4). Ferri-form signals were also detected, but these signals revealed even smaller absolute intensities.

A similar finding was observed with the potassiated desferri-FC variant (756.2919), which was shaded by phosphocholines extending up to 6 × 10^8^ counts of absolute ion intensity. The absolute intensities of the [FC + Na]^+^ and [FC + K]^+^ ions ranged in the order of 10^6^, whereas the corresponding ferri-forms were notably less abundant (10^4^) (Supplementary Fig. S7).

**Figure 5:**
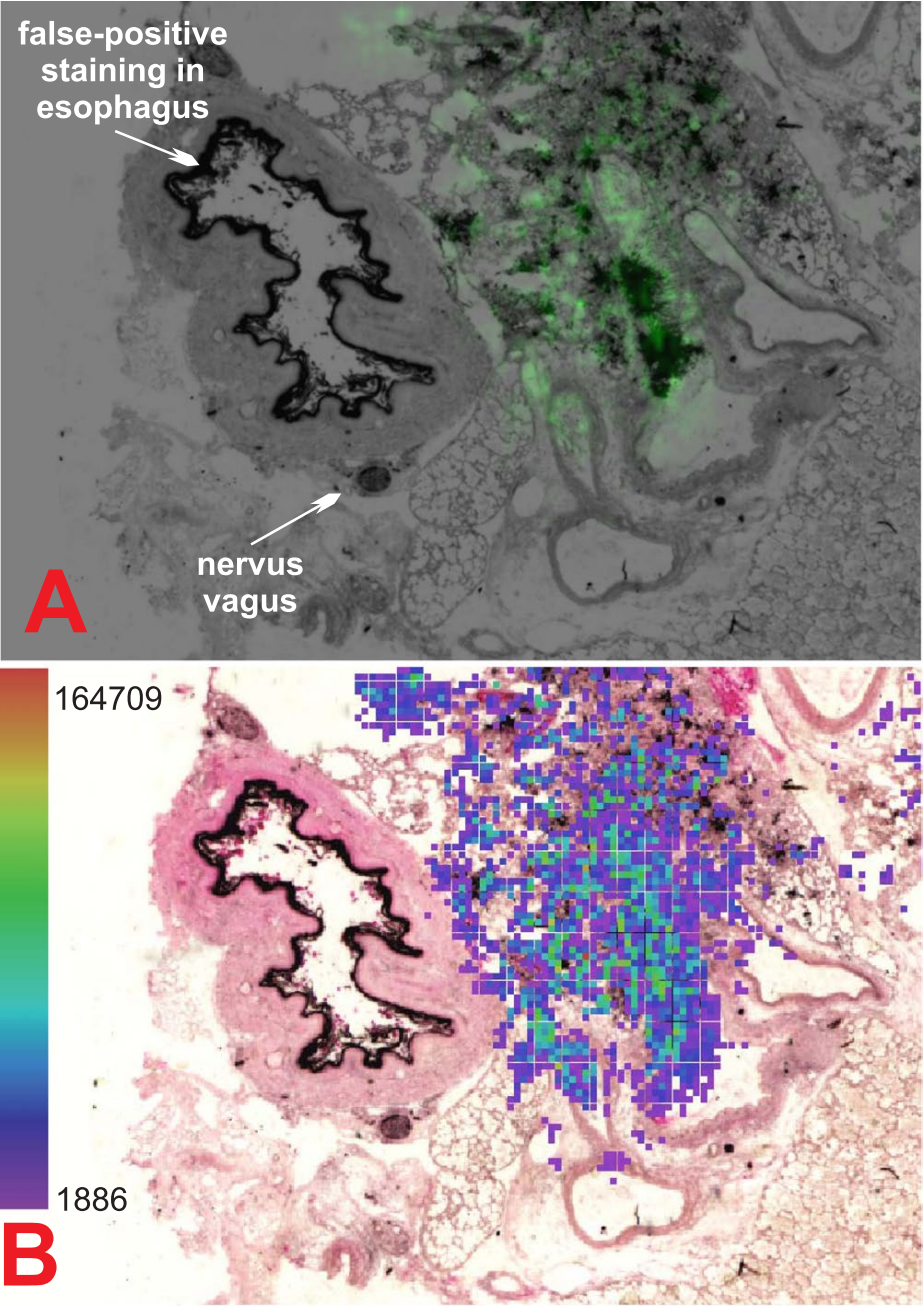
MALDI-CASI-FTICR-MS image (Bruker FlexImaging 4.1) of combined [FC + Na]^+^ and [FC + K]^+^ (±0.001 Da) distribution illuminated in green (**A**). Graphic output of our dereplication tool, i.e., CycloBranch, indicating the annotation of the siderophores (**B**)^18^.

The highest FC concentrations correlated well with areas with massive fungal burden as indicated by the histology analysis (Fig. 1). FC was also detected by MSI in areas that were not histologically predicted to be infected (Supplementary Fig. S7). Therefore, mass spectrometry has a higher sensitivity than optical microscopy. Furthermore, GMS staining can lead to false-positive results. For instance, a polysaccharide structure was detected in the esophagus. Although these complex sugars were labeled in black by GMS, they were not markers of fungal infection (Fig. 5, top). The esophagus is not embedded into the lungs but is located parallel to the trachea. In this case, the lungs were removed from the animal along with the esophagus, and upon tissue freezing, both tissue specimens remained together.

The presence of FC was inferred from both exact and product mass spectra measurements. MALDI tandem mass spectrometry (MS/MS) of the infected tissue provided the same product ions as those reported for the standard sodiated FC (Supplementary Fig. S8). These spectra revealed typical losses of C_2_H_4_O_2_ units^14^. For additional characterization, we performed a dereplication of the microbial secondary metabolites provided by our in-house software, i.e., CycloBranch, which uses an integrated library of fungal secondary metabolites^18^. For this analysis, we implemented a new multimodal imaging module that enabled us to fuse imaging mass spectrometry data with histology and optical scans of the indium-tin oxide (ITO) MALDI plate. Thus, relative siderophore distributions in the tissue sections were observed (Fig. 5, bottom). This open-source, cross-platform, stand-alone application, which was originally developed for the *de novo* sequencing of non-ribosomal peptides^17^, was recently applied to the *de novo* sequencing of cyclic and branch-cyclic siderophores containing polyketide building blocks^18^. A typical graphical output of sodiated desferri-FC sequenced *de novo* is illustrated in Supplementary Fig. S9. This software can be downloaded for free at http://ms.biomed.cas.cz/cyclobranch.

Finally, the extent of invasive aspergillosis in bulk tissue sections was also evaluated by quantitative mass spectrometry (Supplementary Fig. S10). The concentration of FC was determined in four infected animals (L1, L4, L11, L12); the FC concentration oscillated in the ppm range (mean 13.9 ± 7.5 μg/g and median 9.2 μg/g, dry weight), whereas the TAFC concentration was below the LOD. In poly[N-(2-hydroxypropyl)methacrylamide] (pHPMA)-filled lungs (L1, L4), the concentration of the siderophores was lower than that in intact lungs (L11, L12). Although the pHPMA polymer has been reported to be suitable for the ionization of peptides and proteins by MALDI^23^, in our siderophore application, we noticed interference resulting in siderophore signal reduction by approximately one order of magnitude. No siderophores were detected in the control animals.

## Materials and Methods

### Experimental model of aspergillosis in rats

Both the *Aspergillus* cultivation procedures and the experimental model of aspergillosis have been described in detail elsewhere^13^. Briefly, *A. fumigatus* 1059 CCF was obtained from the Culture Collection of Fungi, Faculty of Science, Charles University in Prague and maintained on yeast medium slants (0.3% malt extract, 0.3% yeast extract, 0.5% peptone, and 0.5% glucose) at 4 C°. All animal experiments were conducted in accordance with the regulations and guidelines of the Czech Animal Protection Act (No. 246/1992) with the approval of the Czech Ministry of Education, Youth and Sports (MSMT-21235/2013-12) and the institutional Animal Welfare Committee of the Faculty of Medicine and Dentistry of Palacky University in Olomouc, Czech Republic. The studies were performed using two groups of female Lewis rats (2–3 months old, Harlan Laboratories). The first group (twelve animals) included immunocompromised rats infected with *Aspergillus*. The second (control) group included three immunocompromised animals.

To prevent the animals from developing bacterial super-infection, all fifteen animals (twelve infected and three controls) were treated with 2 mM ciprofloxacin (Ciprofloxacin Kabi, Fresenius Kabi, Czech Republic) and 0.1 mM polymyxin E (Colomycin, Forest Laboratories, UK) in their drinking water throughout the experiment. Five days before the inoculation, the first group received the immunosuppressive DNA-alkylating agent cyclophosphamide (Endoxan, Baxter, Czech Republic, i.p., 75 mg/kg) and the antibiotic teicoplanin (Targocid, Sanofi, Czech Republic, i.m., 35 mg/kg). One day before the infection, the administration of both drugs was repeated with the following modification: the teicoplanin dose was reduced to 25 mg/kg (i.m.). On the day of inoculation, the administration of teicoplanin was repeated (25 mg/kg, i.m.), followed by an intratracheal application of *Aspergillus* spores (100 μL, 10^8^ CFU/mL). The experiment was concluded (day 3 post-infection) based on the monitoring of the health status of each animal and our previous experience with a rat model of *Aspergillus* infection^24^. The control animals underwent the same treatment as the infected animals, except for the administration of the *Aspergillus* spores.

Six animals that were infected with *Aspergillus* died before day 3 post-infection and were excluded from the experiment. At the end of the experiment, the nine surviving animals were sacrificed by an overdose of a ketamine (Calypsol, Gedeon Richter, Hungary) and xylazine (Xylazin Ecuphar, Ecuphar, Germany) mixture (2:1). Three lung sample sets (L-samples) were compared as follows: collapsed infected lungs (L2, L11, L12), infected lungs inflated with the pHPMA polymer (L1, L4, L8)^23^, and lungs from control animals dosed only with the immunosuppressants (L13 with polymer, L14, L15). At the end of the experiment, the urine (U-samples) and serum (S-samples) were collected in parallel from all nine survivors.

### Statistical analysis

The diversity in the technical replicates was measured by the coefficient of variation (Supplementary Table S1). The heterogeneity in the biological replicates is presented as the mean ± standard error of the mean and was assessed by the coefficient of variation (Supplementary Tables S2 and S3). Gaussian distribution of data sets was assessed by Jarque-Bera tests and Lilliefors corrected Kolmogorov-Smirnov tests (Supplementary Table S4). The normality assumption was not rejected, so the parametric statistics were used. The statistical analyses were performed using one-tailed z-tests under the null hypothesis that the Fe-TAFC, Fe-FC and iron concentrations were under the detection limit (Supplementary Table S5). The pair differences within all three groups (Fe-TAFC, Fe-FC, iron) were subjected to the standard Student’s two-sample t-test to determine the statistical significance of the detected changes (Supplementary Table S6 and Fig. S11). The significance level was set at *p* < 0.05. Statistical analysis was performed using Matlab software (MATLAB version 8.6 (R2015b), Natick, Massachusetts: The MathWorks Inc.).

### Extraction, separation and quantification of the siderophores

The siderophore extractions from urine, serum and lung tissues were performed according to the adapted protocol for metabolite profiling^25^. Briefly, the urine samples (100 μL) were diluted with methanol (400 μL) to provide an approximately 80% final methanolic solution. The samples were gently shaken and incubated for 8 h at −80 °C. Then, the sample was centrifuged at 14,000 × *g* for 10 min (4–8 °C), and the supernatant was transferred to a new 1.5 mL microcentrifuge tube, lyophilized to dryness and stored at −80 °C. The serum samples were treated in the same way with the following modification: the sera (200 μL) were centrifuged in Amicon 3 kDa ultra centrifugal filters (Millipore Sigma, Darmstadt, Germany). The third remaining sample type, the bulk lung tissues (19–50 mg) from the four infected and one control animal, was collected, dried and precisely weighed. Each sample was homogenized by milling with glass beads (1 mm diameter) in methanol (1 h). The supernatant was collected, the beads were decanted with methanol twice, and the extracts were pooled and dried.

Prior to the LC/MS analyses in triplicates, all samples prepared by extraction as indicated above were re-dissolved in water (50 μL) and loaded onto an Isolute ENV + SPE column (Labicom, Olomouc, Czech Republic). The charged contaminants were eluted with 50 mM ammonium acetate (pH 5.3), and the components of interest were extracted with 0.5 mL of methanol. The solutions were then dried and dissolved in 5% acetonitrile (100 μL) as the starting solvent. Each sample was loaded (20 μL loop) onto a BEH C18/1.7 μm, 2.1 × 5 mm VanGuard precolumn (Waters, Prague, Czech Republic) at a 50 μL/min flow rate. The siderophores were then separated on analogous analytical columns (1 × 150 mm) with the following gradient: 0–2 min (2% B), 35 min (60% B), 38–40 min (95% B), and 41–50 min (2% B), where A and B represent 0.1% formic acid in water and 0.1% formic acid in acetonitrile, respectively.

The positive-ion ESI mass spectra were collected online using a 12 T SolariX FTICR mass spectrometer (Bruker Daltonics, Billerica, MA, USA). The ion source was operated at 4,500 V with drying gas (N_2_) at a 5 L/min flow rate. The end-plate offset was adjusted to −200 V. The collision voltage and time-of-flight values were −5 V and 1.2 ms, respectively. The mass spectra were collected in the CASI mode (700–1,000 m/z window) at an approximately 0.3 Hz frequency with a mass accuracy better than 3 ppm. The extracted ion chromatograms with 0.01 Da spectral width were used for the integration.

The siderophores were quantified using external matrix-matched calibration standards (EMC Microcollections GmbH, Tübingen, Germany) prepared from 0.1 ng/mL to 10 μg/mL. The Fe-TAFC and Fe-FC ranges were quantified by a weighted linear regression (1/*x* ^2^) via three technical replicates according to the literature^26^. The LOD and LOQ were calculated as 3.3 × (SD/slope) and 10 × (SD/slope) in 0.1–100 ng/mL concentration intervals (SD represents the Standard Deviation), respectively. The responses were summed from the integrated areas of the ferri-forms of protonated, sodiated and potassiated species, which are represented by [M + Fe-2H]^+^ , [M + Fe + Na-3H]^+^ , and [M + Fe + K-3H]^+^ ions, respectively. The acquired spectra were analyzed using DataAnalysis 4.4 (Bruker Daltonics, Germany), converted using CompassXport 3.0 (Bruker Daltonics, Bremen, Germany) and processed with CycloBranch^17,18^.

### Lung cryosectioning, histological staining and MALDI imaging with semi-quantification

The deeply frozen native infected and control lung tissue samples were allowed to warm to −13 °C prior to sectioning with a Leica cryomicrotome CM1950 (Leica, Germany). The tissues were cut into 15 μm-thick slices and mounted onto pre-cooled ITO glass slides (Bruker Daltonics, Bremen, Germany) for the MALDI-MSI experiments in at least two technical replicates. Consecutive slices used for the optical microscopy were thaw-mounted. Both slide types were vacuum-dried in a desiccator at room temperature for 40 min. Using a modified GMS staining procedure (Sigma-Aldrich kit, Germany), the fungal polysaccharide wall was oxidized with periodic acid to release the aldehyde groups. At a basic pH, these groups reacted with methenamine silver in the impregnation step, reducing it to metallic silver and rendering the fungal cells visible. The redundant silver was removed with a sodium thiosulfate solution. The tissue sample was then counterstained with Eosin Y (VWR Chemicals, Czech Republic), dehydrated using stepwise (95 and 100%) concentrations of ethanol, toned in xylene and fixed with DPX medium (Sigma-Aldrich, Germany). Alternatively, hematoxylin and eosin staining was used for the standard histopathological evaluation. In this case, the specimens were rehydrated, stained with hematoxylin Gill no. 2 (Sigma Aldrich, Czech Republic), flushed with water, and the excessive colorant was removed with acidic ethanol. Subsequently, eosin staining was performed. The same procedure was repeated as in the previous case. All specimens were evaluated under a DN45 light microscope (Lambda Prague, Prague, Czech Republic) equipped with a Canon EOS 700D camera.

The MALDI-MSI was performed using the same FTICR mass spectrometer in the CASI mode with a quadrupole-narrowing window in the 700–1,100 mass range. The matrices α-cyano-4-hydroxy-cinnamic acid (CHCA, 10 mg/mL in 50% ACN/0.1% TFA) and dihydroxybenzoic acid (DHB, 30 mg/mL in 50% ACN/0.1% TFA) were mixed at a 1:1 ratio and applied to the prepared tissues using an ImagePrep (Bruker Daltonics, Bremen, Germany, default method for CHCA) device. The siderophores were desorbed with a 50 μm raster step size using a SmartBeam II laser (2 kHz, 200 shots/pixel) and 320,000 resolution (FWHM) at m/z 400. The product ion mass spectra were collected at a 3 Da isolation width and 30 V collision energy. Desorption from the pure standards or tissues was performed using laser attenuations of 22% or 26%, respectively. The raw mass spectra were exported as an imzML file using FlexImaging 4.1 (Bruker Daltonics, Bremen, Germany) and batch-processed using CycloBranch^18^ against the database of siderophores^11^. The product ion mass spectra were evaluated using DataAnalysis 4.4 (Bruker Daltonics, Bremen, Germany) software.

In this work, the siderophores are reported in both ferri- or desferri-forms. Siderophore ferri-protonated, ferri-sodiated and ferri-potassiated species are represented by [M + Fe-2H]^+^ , [M + Fe + Na-3H]^+^ , and [M + Fe + K-3H]^+^ , respectively. Analogous desferri-forms are reported as [M + H]^+^ , [M + Na]^+^ , and [M + K]^+^ , ions. The molecular descriptor M may represent any siderophore (FC or TAFC).

### ICP-MS

The concentration of iron bound to the siderophores was quantified in the urine by solution ICP-MS. The chemicals, reagents, procedures and instrumentation have been previously reported elsewhere^13^. In the urine samples, the free iron was removed by solid phase extraction on an Isolute ENV + SPE cartridge (Biotage, Olomouc, Czech Republic). The eluents were then precisely diluted with 1% (v/v) nitric acid to 3 mL. The iron content in the 0.1 to 10 μg/L range was measured using an Agilent 7700x ORS-ICP mass spectrometer (Agilent Technologies, Japan) equipped with an ASX-520 autosampler and octopole reaction system working in helium mode to overcome the spectral interferences observed on ^56^Fe and ^57^Fe isotopes.

### Data availability

All data generated or analyzed in this study are included in this published article and its supplementary information files. Original raw datafiles can be downloaded from this persistent web link: https://owncloud.cesnet.cz/index.php/s/GzxKD094iYVQbyr.

## Conclusions

*Aspergillus fumigatus* produces a diverse set of mycotoxins and secondary metabolites that can be used as disease biomarkers in an infected host. The spectrum of these microbial products is dependent on the *Aspergillus* species and on the cultivation or stress conditions. The excretion of fungal virulence factors is expected to be high during the early stages of infection. For example, gliotoxin^9^ or fungal siderophore production is essential for *Aspergillus* strain virulence^12^. Because siderophores are not synthesized by mammalian hosts, the detection of fungal metabolites in an infected host is a valid diagnostic assay rather than simply a confirmatory approach.

In this work, we identified two microbial siderophores, i.e., FC and TAFC, as markers of IPA infection that can be non-invasively detected in urine from an infected host. These molecules can be detected in the ferri- or desferri-forms. In addition, their combinations with sodium and potassium ions were also observed in the serum and lung tissue samples, making siderophore quantification more challenging. We provided robust qualitative and quantitative data indicating the strong analytical potential of the CASI-FTICR approach. The best results were obtained in the HPLC-ESI-CASI-FTICR experiments. The LODs of Fe-TAFC and Fe-FC in the rat serum were 0.28 and 0.36 ng/mL, respectively. In the rat urine the respective limits of detection were lower and achieved 0.02 and 0.03 ng/mL. In the infected animals, ferricrocin concentrations in the rat sera fluctuated in the 3–32 ng/mL range. Importantly, the biomarker concentrations were much higher in the urine and could reach up to 1 μg/mL. The siderophore concentration in the bulk lung tissues was in the ppm range (μg of siderophore/g of tissue). In the tissue assessment, the molecular specificity of MSI could outperform optical microscopy with GMS staining, clarifying its false-positive and false-negative results. Furthermore, the iron concentration (siderophore-bound) in the urine from infected animals was approximately 17 × higher than that in the controls (assessed in the solution ICP-MS experiments). In contrast to the less-specific bound iron detection, the molecular quantification has a much better specificity as the siderophores revealed no or little chemical background.

TAFC and FC are panfungal markers, as their production was described with some other microbial genera. e.g. *Fusarium graminearum, Fusarium sacchari, Paracoccidioides* or *Pseudogymnoascus* species^27,28^. On the contrary, namely *A. fumigatus* and *A. nidulans* could be considered as important siderophore producers that are most clinically relevant^29^. Galactomannan^30^ as well as 1,3-*β*-D-glucan tests^31^ may exhibit poor positive predictive value (Supplementary Fig. S2). The low limit of detection of TAFC and FC in serum or urine and high positive predictive value of the test based on siderophores may improve the overall diagnostic accuracy of aspergillosis detection, either alone or in concert with other non-invasive or invasive diagnostic armory.

## Electronic supplementary material

Supplementary information
