# Supplementary material for: Non-invasive and invasive diagnoses of aspergillosis in a rat model by mass spectrometry
Source: Sci Rep. 2017 Nov 28;7:16523. doi: 10.1038/s41598-017-16648-z (PMC5705710; doi:10.1038/s41598-017-16648-z)
Supplement: Supplementary file 1 — Supplementary information [file 41598_2017_16648_MOESM1_ESM.pdf]

Non-invasive and invasive diagnoses of aspergillosis in a rat model by mass spectrometry

Dominika Luptáková[1,+], Tomáš Pluháček[1,2,+], Miloš Petřík[3], Jiří Novák[1], Andrea Palyzová[1], Lucie Sokolová[1], Anton Škríba[1], Blanka Šedivá[1,4], Karel Lemr[1,2], and Vladimír Havlíček[1,2,*]

[1]Institute of Microbiology of the Czech Academy of Sciences, Prague 4, 142 20, Czech Republic
[2]Regional Centre of Advanced Technologies and Materials, Department of Analytical Chemistry, Olomouc, 771 47, Czech Republic
[3]Institute of Molecular and Translational Medicine, Palacky University, Olomouc, 779 00, Czech Republic
[4]University of West Bohemia, Plzen, 306 14, Czech Republic
[*]vlhavlic@biomed.cas.cz
[+]These authors contributed equally to this work

Table of contents

Figure S1. Coronal slices (CT and PET/CT) and 3D volume rendered (PET/CT) images of infected and control animals dosed with radioactive ^{68}Ga-TAFC. PET/CT images were acquired 45 min post injection.

Figure S2. Distribution of Fe-FC in infected (S1, S2, S4, S8, S11 and S12) and control (S13-S15) samples of rat sera (Panel A). ND stands for ”not detected”. Optical density indexes obtained from galactomannan testing (Platelia Aspergillus EIA, BioRad) on identical serum samples (Panel B). The mutual test performance comparison among three detection systems applied on serum samples: Fe-FC concentration [ng/mL] by LC-MS vers. 1,3-β-D-glucan concentration [pg/mL] vers. galactomannan test [ODI=optical density index]. 1,3-β-D-glucan and galactomannan test were from Fungitell (Associates of Cape Cod, Falmouth, USA) or BioRad (Bio-rad, Prague, Czech Republic), respectively (Panel C).

Figure S3. Calibration curves for Fe-TAFC in the rat serum (top), urine (middle) and tissue (bottom). The LOD and LOQ in the rat serum were determined as 0.28 and 0.85 ng/mL, respectively, using linear regression with weight function $1/x^2$ in 0.1 - 100 ng/mL concentration interval. $y = x \times 10^7 - 9.8 \times 10^6$, $R = 0.999$. In the rat urine and tissue the respective LODs and LOQs were lower and achieved 0.02 and 0.05 ng/mL for urine and 0.04 and 0.12 ng/mL for tissue, respectively, using linear regression with weight function $1/x^2$ in 0.1 - 10 ng/mL concentration interval. $y = 2.4x \times 10^6 + 0.9 \times 10^6$, $R = 0.986$ (urine) and $y = 2.9x \times 10^6 + 3.1 \times 10^6$, $R = 0.991$ (tissue).

Figure S4. Calibration curves for Fe-FC in rat serum (top), urine (middle) and tissue (bottom). The LOD and LOQ in the rat serum were determined as 0.36 and 1.08 ng/mL, respectively, using linear regression with weight function $1/x^2$ in 0.1 - 100 ng/mL concentration interval. $y = 4.4x \times 10^6 - 2.2 \times 10^6$, $R = 0.997$. In the rat urine and tissue the respective LODs and LOQs were lower and achieved 0.03 and 0.08 ng/mL, respectively, using linear regression with weight function $1/x^2$ in 0.1 - 10 ng/mL concentration interval. $y = 1.2x \times 10^6 + 1.2 \times 10^6$, $R = 0.953$ (urine) and $y = 2x \times 10^6 + 10^6$, $R = 0.985$ (tissue).

Figure S5. Extracted ion (10 mDa window) mass chromatograms of $[TAFC+Fe-2H]^+$, $[TAFC+Fe+Na-3H]^+$, and $[FC+Fe-2H]^+$ ions represented here by traces 906, 928 and 771, respectively (top). The chromatograms are attributed to the most diluted point (0.1 ng/mL) used for calibration curve construction. The obtained ESI mass spectrum agreed with in silico isotopic profile generated for sodiated Fe-TAFC species $[C_{39}H_{57}N_6O_{15}FeNa]^+$ (bottom).

Figure S6. The signal of $[FC+K]^+$ buried in lung lipids (A) and simulation of the corresponding $[C_{28}H_{47}N_9O_{13}K]^+$ isotope pattern (B).

Figure S7. MALDI MSI revealed lower abundance of $[FC+Fe+Na-3H]^+$ and $[FC+Fe+K-3H]^+$ ions (m/z 793 and 809) compared to the corresponding desferri-forms (m/z 740 and 756). Siderophore signals are shown either alone (all left panels) or fused with optical scans with GMS staining (all right panels).

Figure S8. Product ion MALDI mass spectra of the $[FC+Na]^+$ ion. (A) FC standard received from EMC Microcollections, GmbH. (B) Product ion scan of m/z 740.3157 performed on an infected tissue section. A wider isolation window (3 Da) permitted the dissociation of interfering contaminants having their m/z value close to the siderophore parent.

Figure S9. Screenshot of the *de novo* sequencing result on $[FC+Na]^+$ returned by CycloBranch. For the graphics description, please, see the webpage http://ms.biomed.cas.cz/cyclobranch.

Figure S10. Bulk quantitation of Fe-FC in tissue sections of four infected rats. Note the possible siderophore signal interference with the pHPMA polymer (L1, L4). ND stands for ”not detected”.

Figure S11. The boxplots for infected groups. For the data see the Supplementary Tables.

Table S1. The variabilities in technical replication measured by the coefficient of variation (CV).

Table S2. The variabilities in biological replicates measured by the coefficient of variation (CV) in six infected animals. SEM is the standard error of the mean.

Table S3. The variabilities in biological replication measured by the coefficient of variation (CV) in three control animals. SEM is the standard error of the mean.

Table S4. Skewness, kurtosis, and p-values of normality tests for infected groups. Gaussian distribution of data sets was assessed by Jarque-Bera tests and Lilliefors corrected Kolmogorov-Smirnov tests.

Table S5. The statistical analyses performed using one-tailed z-tests under the null hypothesis claiming that the expected value is under the limit of detection (NAN stands for **N**ot **A** **N**umber).

Table S6. The p-values returned by a standard Student's two-sample t-test.

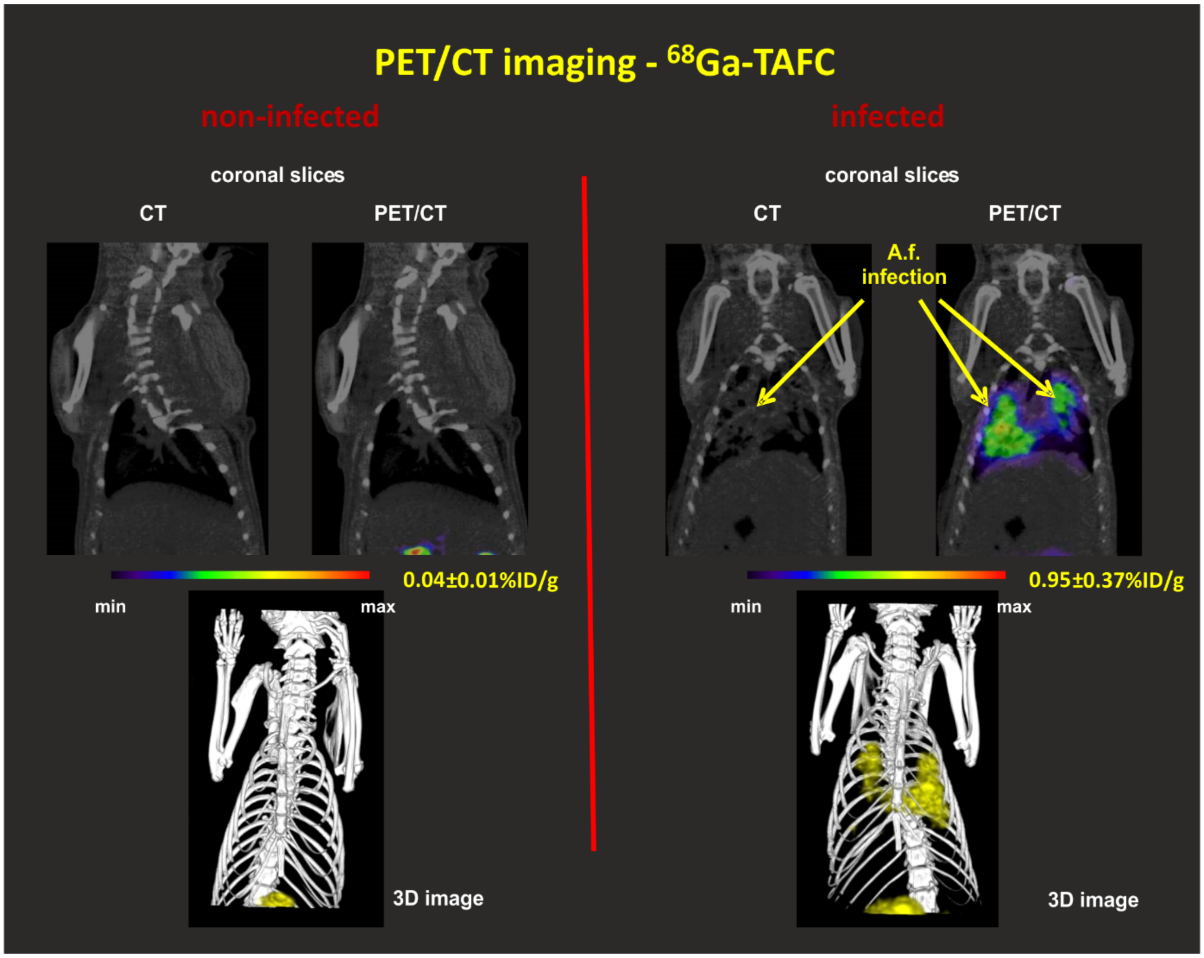

Figure S1. Coronal slices (CT and PET/CT) and 3D volume rendered (PET/CT) images of infected and control animals dosed with radioactive ^{68}Ga-TAFC. PET/CT images were acquired 45 min post injection.

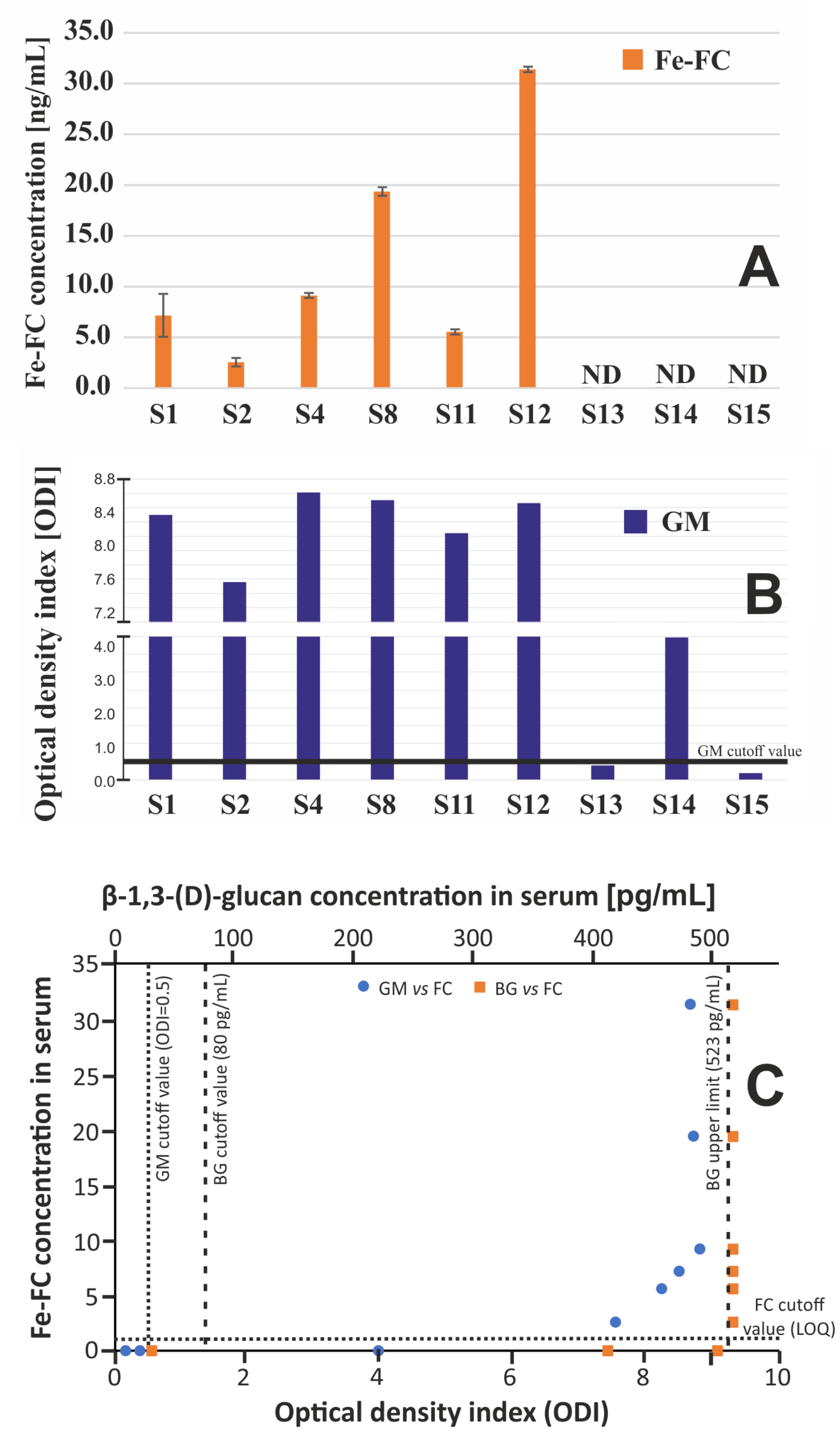

Figure S2. Distribution of Fe-FC in infected (S1, S2, S4, S8, S11 and S12) and control (S13-S15) samples of rat sera (Panel A). ND stands for ”not detected”. Optical density indexes obtained from galactomannan testing (Platelia Aspergillus EIA, BioRad) on identical serum samples (Panel B). The mutual test performance comparison among three detection systems applied on serum samples: Fe-FC concentration [ng/mL] by LC-MS vers. 1,3-β-D-glucan concentration [pg/mL] vers. galactomannan test [ODI=optical density index]. 1,3-β-D-glucan and galactomannan test were from Fungitell (Associates of Cape Cod, Falmouth, USA) or BioRad (Bio-rad, Prague, Czech Republic), respectively (Panel C).

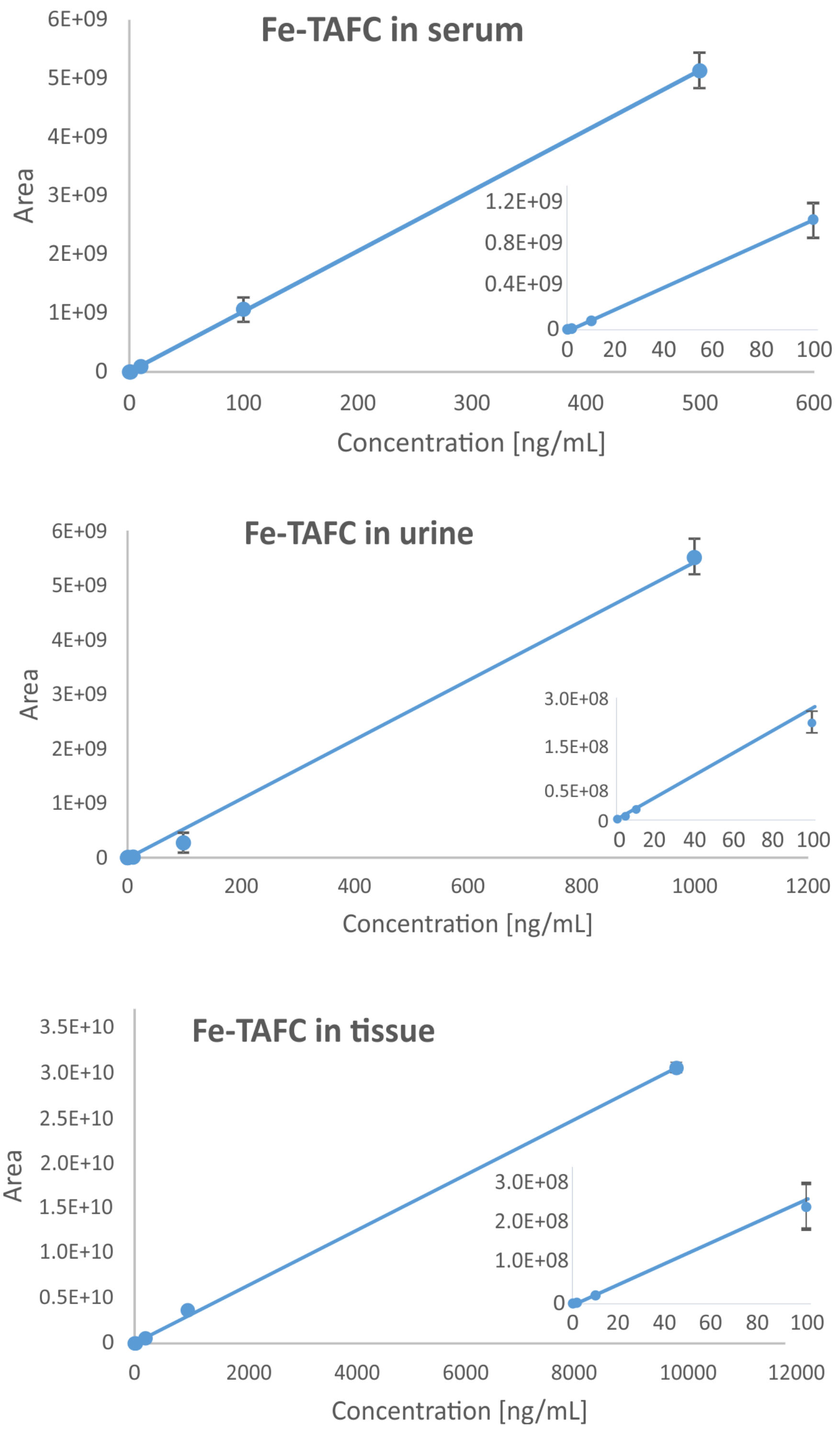

Figure S3. Calibration curves for Fe-TAFC in the rat serum (top), urine (middle) and tissue (bottom). The LOD and LOQ in the rat serum were determined as 0.28 and 0.85 ng/mL, respectively, using linear regression with weight function $1/x^2$ in 0.1 - 100 ng/mL concentration interval. $y = x \times 10^7 - 9.8 \times 10^6$, $R = 0.999$. In the rat urine and tissue the respective LODs and LOQs were lower and achieved 0.02 and 0.05 ng/mL for urine and 0.04 and 0.12 ng/mL for tissue, respectively, using linear regression with weight function $1/x^2$ in 0.1 - 10 ng/mL concentration interval. $y = 2.4x \times 10^6 + 0.9 \times 10^6$, $R = 0.986$ (urine) and $y = 2.9x \times 10^6 + 3.1 \times 10^6$, $R = 0.991$ (tissue).

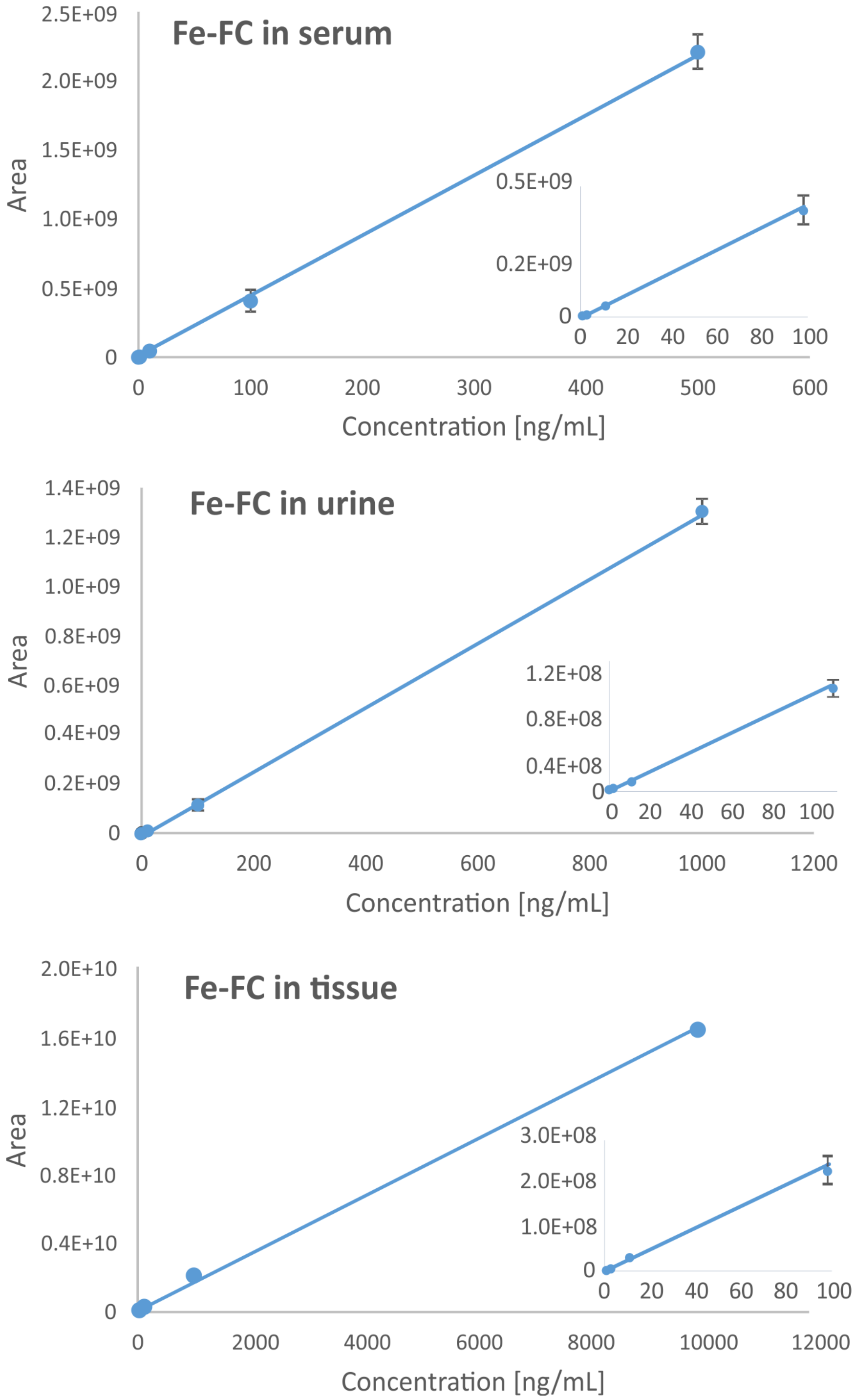

Figure S4. Calibration curves for Fe-FC in rat serum (top), urine (middle) and tissue (bottom). The LOD and LOQ in the rat serum were determined as 0.36 and 1.08 ng/mL, respectively, using linear regression with weight function $1/x^2$ in 0.1 - 100 ng/mL concentration interval. $y = 4.4x \times 10^6 - 2.2 \times 10^6$, $R = 0.997$. In the rat urine and tissue the respective LODs and LOQs were lower and achieved 0.03 and 0.08 ng/mL, respectively, using linear regression with weight function $1/x^2$ in 0.1 - 10 ng/mL concentration interval. $y = 1.2x \times 10^6 + 1.2 \times 10^6$, $R = 0.953$ (urine) and $y = 2x \times 10^6 + 10^6$, $R = 0.985$ (tissue).

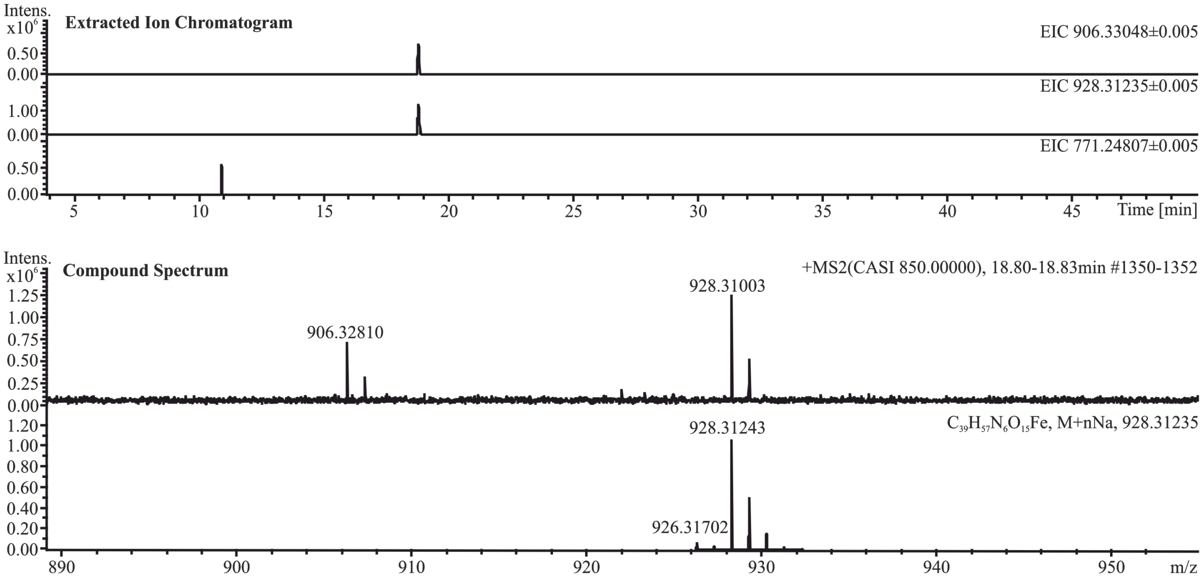

Figure S5. Extracted ion (10 mDa window) mass chromatograms of $[TAFC+Fe-2H]^+$, $[TAFC+Fe+Na-3H]^+$, and $[FC+Fe-2H]^+$ ions represented here by traces 906, 928 and 771, respectively (top). The chromatograms are attributed to the most diluted point (0.1 ng/mL) used for calibration curve construction. The obtained ESI mass spectrum agreed with in silico isotopic profile generated for sodiated Fe-TAFC species $[C_{39}H_{57}N_6O_{15}FeNa]^+$ (bottom).

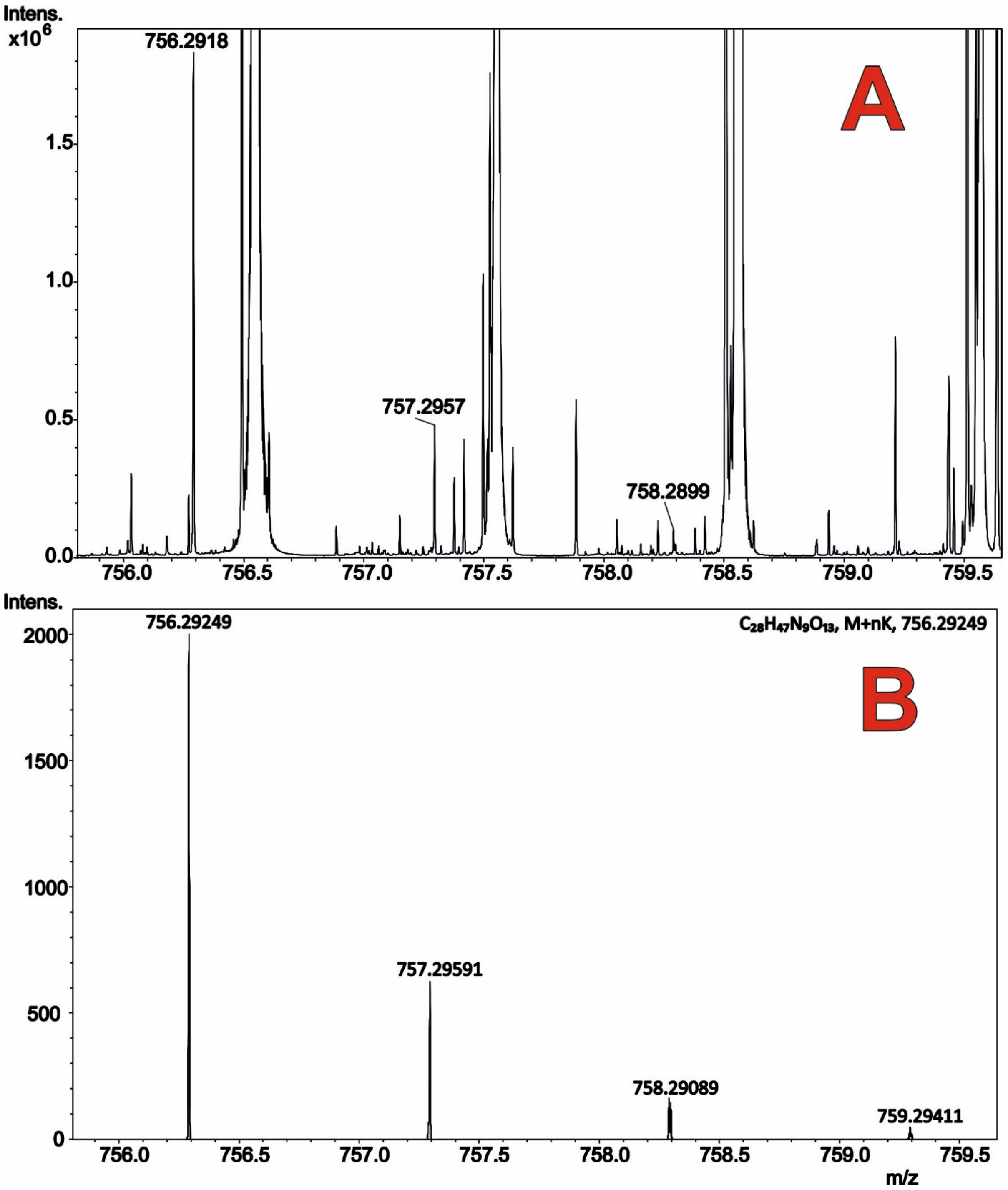

Figure S6. The signal of $[FC+K]^+$ buried in lung lipids (A) and simulation of the corresponding $[C_{28}H_{47}N_9O_{13}K]^+$ isotope pattern (B).

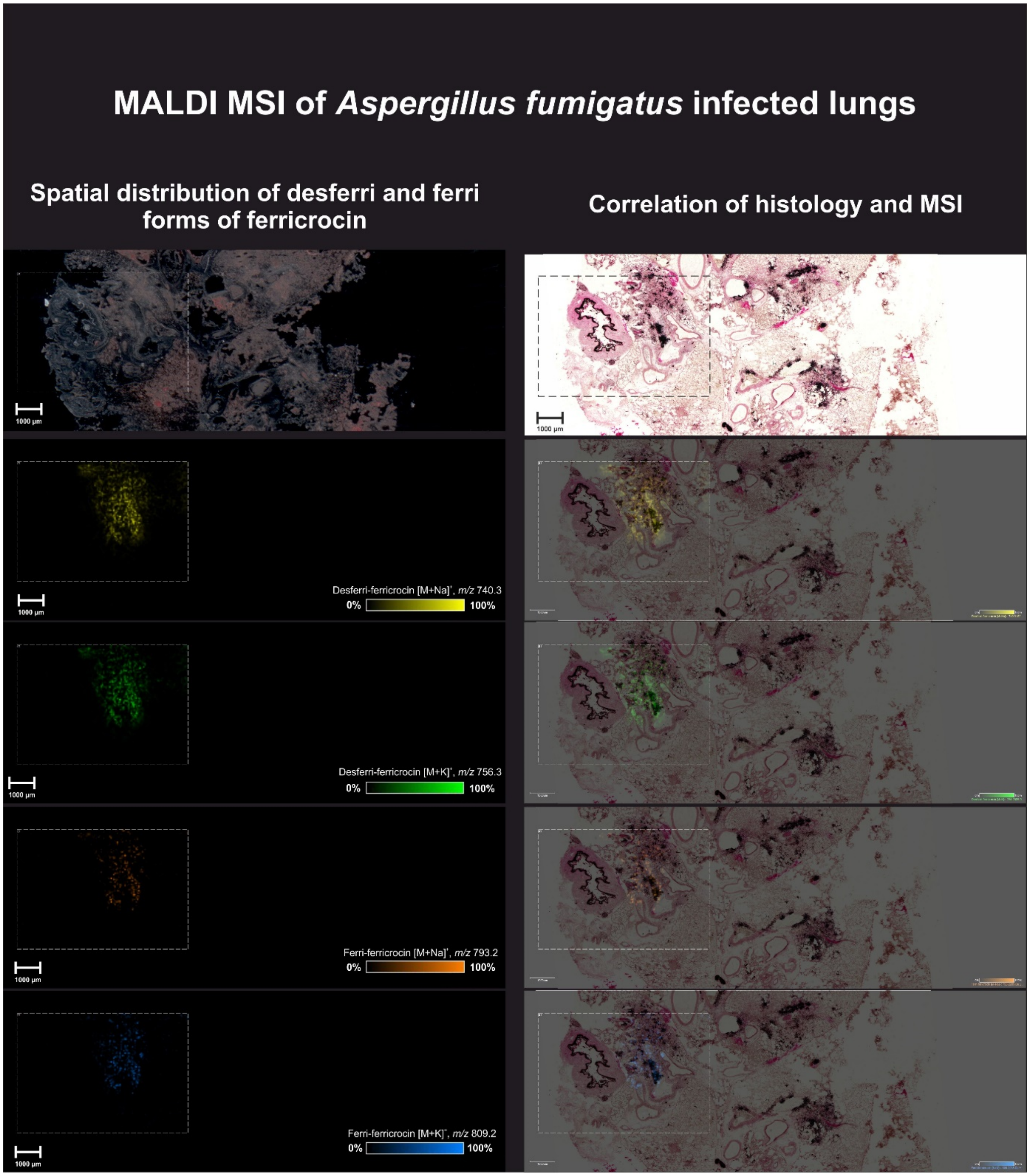

Figure S7. MALDI MSI revealed lower abundance of $[FC+Fe+Na-3H]^+$ and $[FC+Fe+K-3H]^+$ ions (m/z 793 and 809) compared to the corresponding desferri-forms (m/z 740 and 756). Siderophore signals are shown either alone (all left panels) or fused with optical scans with GMS staining (all right panels).

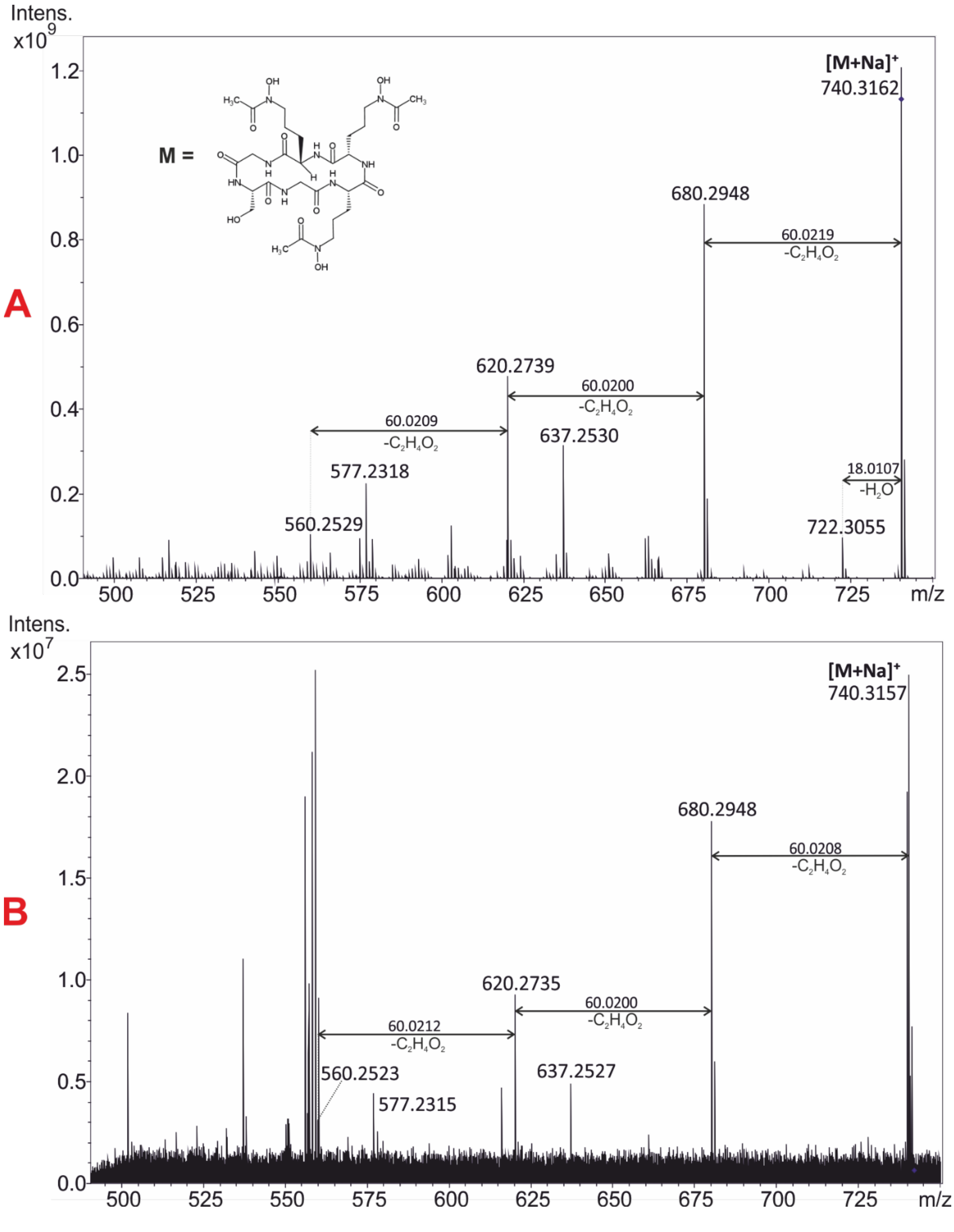

Figure S8. Product ion MALDI mass spectra of the $[FC+Na]^+$ ion. (A) FC standard received from EMC Microcollections, GmbH. (B) Product ion scan of m/z 740.3157 performed on an infected tissue section. A wider isolation window (3 Da) permitted the dissociation of interfering contaminants having their m/z value close to the siderophore parent.

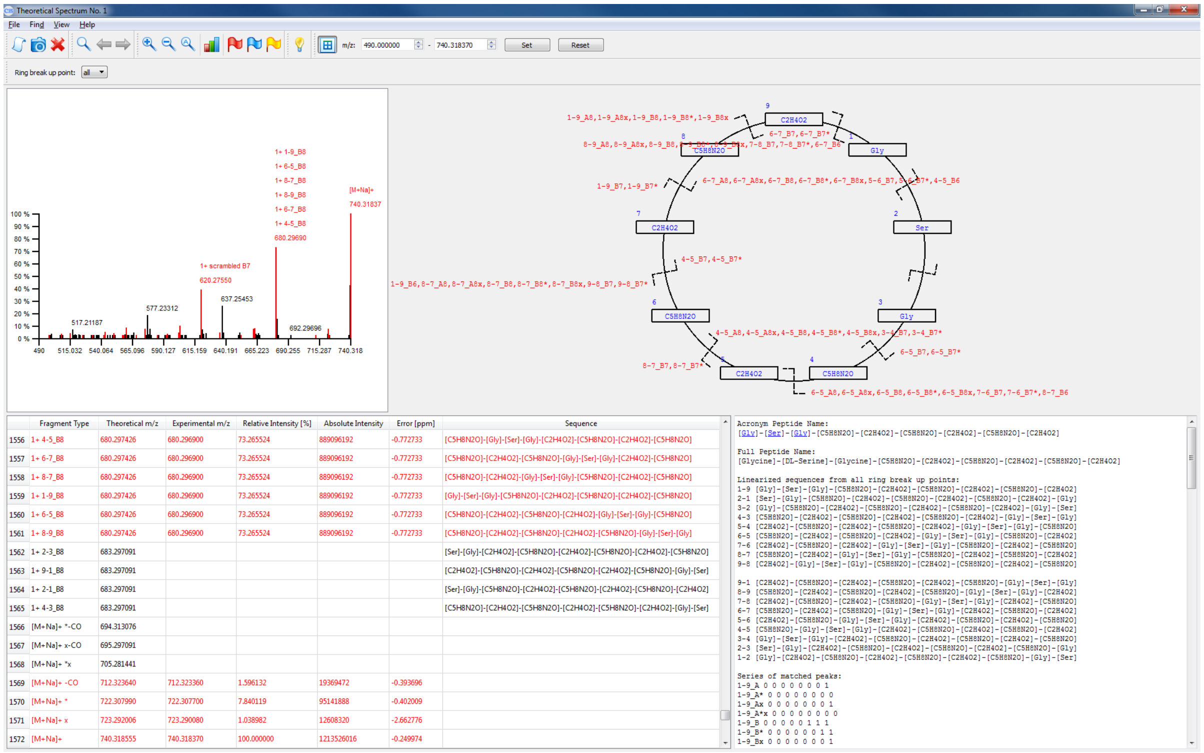

Figure S9. Screenshot of the *de novo* sequencing result on $[FC+Na]^+$ returned by CycloBranch. For the graphics description, please, see the webpage http://ms.biomed.cas.cz/cyclobranch.

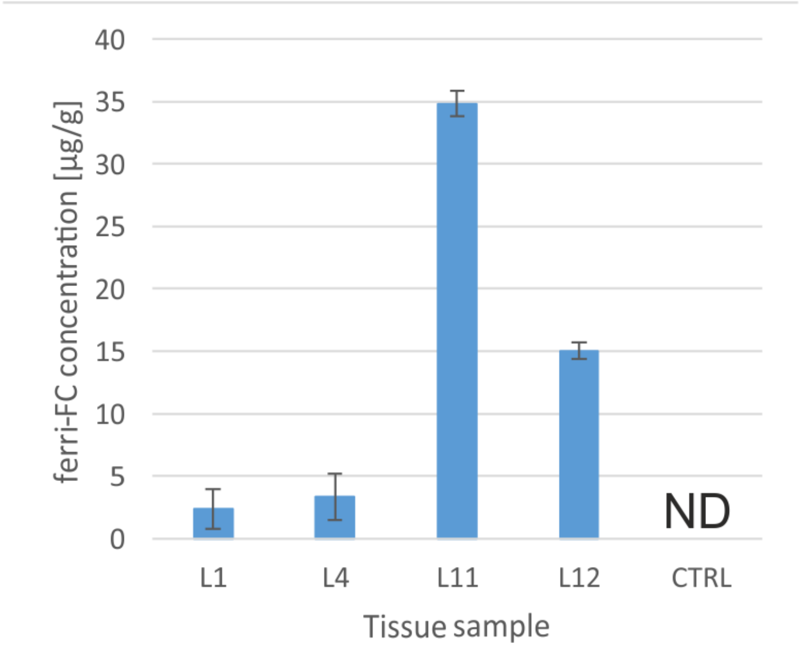

Figure S10. Bulk quantitation of Fe-FC in tissue sections of four infected rats. Note the possible siderophore signal interference with the pHPMA polymer (L1, L4). ND stands for "not detected".

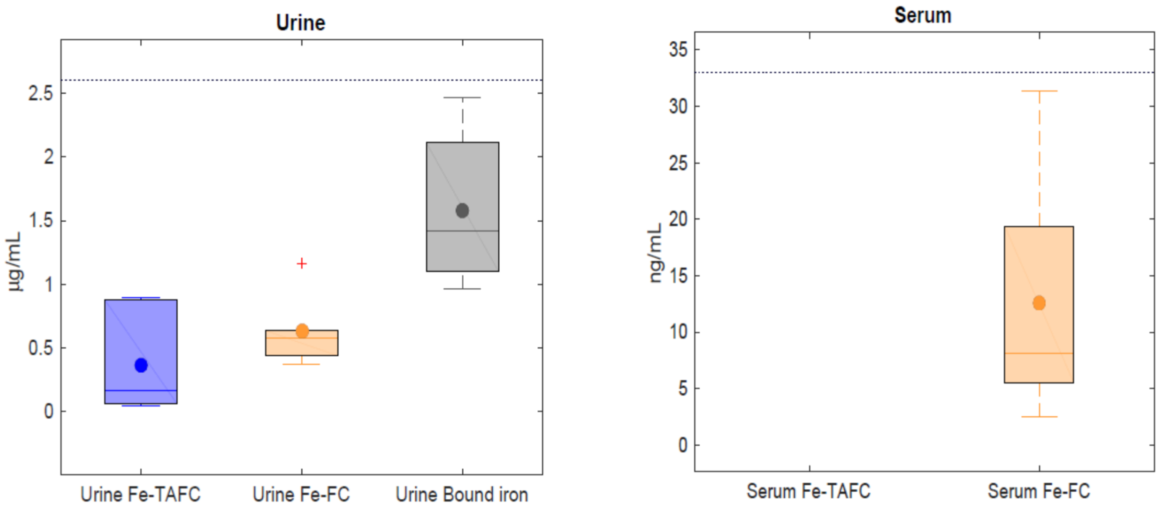

Figure S11. The boxplots for infected groups. For the data see the Supplementary Tables.

Table S1. The variabilities in technical replication measured by the coefficient of variation (CV).

	Mean CV	Standard error of the mean (SEM)
**Urine Fe-TAFC**	0.0333	0.0080
**Urine Fe-FC**	0.0387	0.0045
**Urine bound iron**	0.0693	0.0119
**Serum Fe-FC**	0.0946	0.0467
**Lung Fe-FC**	0.3277	0.1704

Table S2. The variabilities in biological replicates measured by the coefficient of variation (CV) in six infected animals. SEM is the standard error of the mean.

	Limit of detection	Mean	±SEM	CV
**Urine Fe-TAFC**	0.02 ng/mL	0.3674 µg/mL	0.1655 µg/mL	1.1036
**Urine Fe-FC**	0.03 ng/mL	0.6272 µg/mL	0.1154 µg/mL	0.4506
**Urine bound iron**	0.03 µg/mL	1.5784 µg/mL	0.2532 µg/mL	0.3929
**Serum Fe-FC**	0.36 ng/mL	12.5245 ng/mL	4.4407 ng/mL	0.8685
**Lung Fe-FC**	0.36 µg/g	13.8725 µg/g	7.5491 µg/g	1.0884

Table S3. The variabilities in biological replication measured by the coefficient of variation (CV) in three control animals. SEM is the standard error of the mean.

	Mean	±SEM	CV
**Urine bound iron**	0.0930 µg/mL	0.0067 µg/mL	0.1248

Table S4. Skewness, kurtosis, and p-values of normality tests for infected groups. Gaussian distribution of data sets was assessed by Jarque-Bera tests and Lilliefors corrected Kolmogorov-Smirnov tests.

	Skewness	Kurtosis	P-value for Jarque–Bera test	P-value for Lilliefors corrected Kolmogorov-Smirnov test
**Urine Fe-TAFC**	0.6499	1.4997	0.1287	0.0377
**Urine Fe-FC**	1.2600	3.3559	0.0458	0.0530
**Urine Bound iron**	0.3757	1.5390	0.3584	0.1697
**Serum Fe-FC**	0.9390	2.4397	0.1355	0.1216
**Lung Fe-FC**	0.7229	1.8996	0.3267	0.5000

Table S5. The statistical analyses performed using one-tailed z-tests under the null hypothesis claiming that the expected value is under the limit of detection (NAN stands for **N**ot **A** **N**umber).

	P-values	
	**Infected**	**Control**
**Urine Fe-TAFC**	0.0132	NAN
**Urine Fe-FC**	<0.0001	NAN
**Urine Bound iron**	<0.0001	<0.0001
**Serum Fe-FC**	0.0024	NAN
**Lung Fe-FC**	0.0367	NAN

Table S6. The p-values returned by a standard Student's two-sample t-test.

	Metabolite pairs	P-value
**Urine**	Fe-TAFC vers. Fe-FC	0.2304
	Fe-TAFC vers. Bound iron	0.0034
	Fe-FC vers. Bound iron	0.0112
